# Risk of Glaucoma Progression in Patients Using a Calcium Channel Blocker: A Propensity-Matched Cohort Study

**DOI:** 10.1167/iovs.67.5.59

**Published:** 2026-05-21

**Authors:** Ibrahim Qozat, Yazan Abubaker, Pranav Vasu, Isabella V. Wagner, Emily Dorairaj, Mary V. Lang, Darby D. Miller, Syril Dorairaj

**Affiliations:** 1 Department of Ophthalmology, Mayo Clinic, Jacksonville, Florida, United States; 2 Department of Medicine, Creighton University School of Medicine, Phoenix, Arizona, United States; 3 Department of Medicine, Charles E. Schmidt College of Medicine, Boca Raton, Florida, United States; 4 Mayo Clinic Alix School of Medicine, Jacksonville, Florida, United States

**Keywords:** calcium channel blockers, primary open-angle glaucoma, disease progression, propensity score matching

## Abstract

**Purpose:**

To investigate the potential impact of calcium channel blocker (CCB) use on the progression of mild to moderate POAG to the severe stage.

**Methods:**

A retrospective cohort analysis was conducted using the TriNetX United States Collaborative Network to identify patients with mild or moderate POAG and more than 6 months of follow-up data. Patients were stratified by CCB use. The primary outcome was disease progression to severe POAG at any time point after the initial diagnosis, identified using *International Classification of Diseases*, Tenth Revision, code H40.1193. Patients with prior severe POAG or those prescribed angiotensin-converting enzyme inhibitors, angiotensin receptor blockers, or beta-blockers were excluded. Propensity score matching (1:1) was conducted to control for age, sex, race, ethnicity, and hypertension. Risk ratios (RRs) with 95% confidence intervals (CIs) were calculated.

**Results:**

After propensity score matching, 7446 patients were included: 3723 controls (no CCB use), 3039 taking dihydropyridine CCBs (dCCBs), and 684 taking nondihydropyridine CCBs (ndCCBs). The risk of severe POAG was significantly higher in patients using dCCBs (RR, 1.67; 95% CI, 1.23–2.27) and ndCCBs (RR, 3.62; 95% CI, 1.97–6.62) compared with controls.

**Conclusions:**

Both dCCBs and ndCCBs were associated with an increased risk of progression to severe POAG in patients with mild to moderate disease. Further research is needed to elucidate the underlying mechanisms of this association.

Glaucoma is the second leading cause of blindness worldwide after cataracts^1^ and the leading cause of irreversible blindness.^2^ In addition to elevated IOP, risk factors include age and myopia.^3^ Reducing IOP remains the primary treatment strategy, achieved through pharmacotherapy, laser therapy, or surgery, with the goal of minimizing optic nerve damage and decelerating visual field loss.^4^^–^^6^ Various classifications have been proposed for glaucoma grading based on optical coherence tomography and visual fields.^7^^–^^9^ The most commonly used classification is a visual field–based classification, the Hodapp–Parrish–Anderson classification, which was introduced in 1993.^10^ This classification takes into consideration the proximity of visual field defects to the fixation point, in addition to the overall damage.^10^^–^^12^

According to the Hodapp–Parrish–Anderson classification, mild glaucoma has a mean deviation of less than 6 dB with all central points within 5° with more than 15 dB. Moderate glaucoma is classified when the mean deviation is less than 12 dB and no points within 5° with 0 dB. In addition, only one hemifield may have a point with a sensitivity of less than 15 dB. Finally, severe glaucoma is classified when the mean deviation is greater than 12 dB, at least one point within the central 5° has a sensitivity of 0 dB, and points within the central 5° with a sensitivity of less than 15 dB in both hemifields.^10^

Certain medications have been associated with glaucoma, either as potential risk factors or protective agents. Among them, calcium channel blockers (CCBs) are of particular interest.^13^^–^^16^ CCBs regulate calcium ion influx through voltage-gated calcium channels found in both cardiovascular and ocular tissues.^17^^,^^18^ Different types of calcium channels have been described, including high-voltage L-type calcium channels, which are abundant in cardiovascular tissue, and low-voltage–gated T-type calcium channels, which are predominantly present in neurons, but are also found in the sinoatrial (SA) node, atrioventricular (AV) node, and neurosecretory cells of the adrenal cortex and medulla.^17^^–^^19^ Because of their predominance in neurons, inhibition of T-type calcium channels may have a neuroprotective effect through improvement of neuronal microcirculation.^19^^,^^20^

CCBs are classified into dihydropyridines (dCCBs) and nondihydropyridines (ndCCBs) based on their structural differences.^17^ First introduced in the 1960s, CCBs are now widely prescribed for conditions such as angina, hypertension, and coronary artery disease.^18^^,^^21^ Whereas ndCCBs have inhibitory effects on the SA and AV nodes, thereby slowing cardiac conduction and contractility, dCCBs have a more peripheral vasodilator effect, which makes them useful for the treatment of arterial hypertension and coronary artery disease.^22^^,^^23^ The effect of CCBs on glaucoma is debated in the literature. Although some studies suggest that CCBs may increase glaucoma risk,^14^^,^^24^ others propose protective mechanisms, including enhanced optic nerve head blood flow through vascular smooth muscle relaxation and vasodilation,^25^^,^^26^ as well as improved aqueous outflow resulting from L-type calcium channel inhibition, which reduces extracellular matrix deposition in the trabecular meshwork and promotes its relaxation.^27^

Given these conflicting findings, this work aimed to investigate the impact of CCBs on the progression of POAG, the most common glaucoma subtype, from the mild to moderate stage to the severe stage. Understanding this relationship may inform decisions regarding the timing and nature of medical and surgical interventions.

## Methods

### Database

A retrospective cohort study was conducted using the TriNetX network, an electronic health records database from major health care organizations across the United States and internationally. The database includes deidentified data from more than 119 million patients across 80 health care organizations in the United States, Taiwan, Georgia, and Brazil. TriNetX, LLC adheres to the Health Insurance Portability and Accountability Act regulations and holds International Organization for Standardization (ISO) 27001:2013 certification. All data in the platform are presented in deidentified, aggregate form and are therefore exempt from institutional review board approval.

Data for this study were collected on December 1, 2024, from the TriNetX US Collaborative Network, which provided access to electronic medical records, including diagnoses, procedures, medications, laboratory values, and genomic information. Records from January 1, 2004, to August 30, 2024, were reviewed. This study adheres to the Strengthening the Reporting of Observational Studies in Epidemiology guidelines for cohort study reporting.^28^

### Cohorts

Patients with a diagnosis of mild or moderate POAG and orally prescribed CCBs were identified using *International Classification of Diseases*, Tenth Revision (ICD-10), codes (H40.11 for POAG) and RXNorm codes for CCB medications. CCB prescriptions included dCCBs (nisoldipine [7435], nimodipine [7426], isradipine [33910], nicardipine [7396], clevidipine [233603], felodipine [4316], amlodipine [17767], levamlodipine [2376944], and nifedipine [7417]) and ndCCBs (diltiazem [3443] and verapamil [11170]). CCB users were stratified into dCCB and ndCCB subgroups based on the different effects of these drugs. For example, dCCBs have peripheral vasodilator effects that make them useful for the treatment of hypertension, whereas ndCCBs are more useful for the treatment of supraventricular tachycardia because of their effect on the SA and AV nodes.^22^^,^^23^

A control cohort comprised patients with mild to moderate POAG who were not prescribed any CCBs. Inclusion criteria for all cohorts required a follow-up duration exceeding 6 months and the absence of concurrent use of angiotensin-converting enzyme (ACE) inhibitors, angiotensin receptor blockers (ARBs), or beta-adrenergic blocking agents.

### Statistical Analysis

Propensity score matching (PSM) was conducted to balance baseline characteristics between patients with mild to moderate POAG taking CCBs and those not taking CCBs. Matching was performed for age at index, sex, race, ethnicity, and arterial hypertension using TriNetX's built-in analysis platform (1:1 nearest-neighbor greedy matching with a caliper of 0.25 standard deviations). The primary outcome assessed was progression from mild to moderate POAG to severe POAG at any time point post diagnosis, identified using ICD-10 code H40.1193.

Standard descriptive statistics were reported for demographic and clinical variables. Cohort differences were assessed using absolute standardized differences, with values of 0.25 or greater considered significant. Relative risks and 95% confidence intervals (CIs) were calculated. Because person-time and exact event times were unavailable, we report risk ratios (RRs) (not hazards) and did not use Kaplan-Meier methods, which can be biased without aligned follow-up and appropriate handling of competing risks.^29^^,^^30^ All statistical analyses were performed using the TriNetX analytics platform.

## Results

### Demographics

Patient demographics before and after PSM in the dCCB and control cohorts are presented in Table 1. Before PSM, the dCCB cohort included 3050 patients, and the control cohort consisted of 38,663 patients. After PSM, both cohorts included 3039 patients. The average age in the dCCB and control groups was 71.2 ± 11.8 years and 71.3 ± 11.5 years, respectively. Most patients in both groups were female (55.7% and 54.6%, respectively) and White (51.1% and 51.2%, respectively). The rate of essential hypertension was 67% in both cohorts after PSM.

**Table 1.** Demographics of Patients With POAG With and Without dCCB Use Before and After PSM

	POAG With dCCB (*N* = 3050)	POAG Without dCCB (*N* = 38,863)		POAG With dCCB (*N* = 3039)	POAG Without dCCB (*N* = 3039)	
Characteristics	Value Before PSM	Value Before PSM	ASD	Value After PSM	Value After PSM	ASD
Age at index	71.2 ± 11.8	68.5 ± 13.2	0.219	71.2 ± 11.8	71.3 ± 11.5	0.011
Essential hypertension	2064 (67.7)	10,603 (27.3)	0.351	2053 (67.6)	2052 (67.5)	<0.001
Sex						
Female	1703 (55.8)	18,807 (48.4)	0.149	1692 (55.7)	1660 (54.6)	0.021
Male	1158 (38.0)	13,885 (35.7)	0.046	1158 (38.1)	1183 (38.9)	0.017
Race						
Black or African American	1203 (39.4)	6522 (16.8)	0.521	1192 (39.1)	1177 (38.7)	0.010
White	1218 (39.9)	18,047 (48.4)	0.132	1218 (40.1)	1250 (41.1)	0.021
American Indian or Alaska Native	10 (0.3)	415 (1.1)	0.089	10 (0.3)	10 (0.3)	<0.001
Unknown	400 (13.1)	7318 (18.8)	0.157	400 (13.2)	386 (12.7)	0.014
Native Hawaiian or other Pacific Islander	10 (0.3)	3680 (9.5)	0.433	10 (0.3)	10 (0.3)	<0.001
Asian	128 (4.2)	1466 (3.8)	0.022	128 (4.2)	140 (4.6)	0.019
Other	93 (3.0)	1413 (3.6)	0.033	93 (3.1)	80 (2.6)	0.026
Ethnicity						
Unknown	949 (31.1)	17,013 (43.8)	0.264	948 (31.2)	905 (29.8)	0.031
Not Hispanic or Latino	1971 (64.9)	19915 (51.2)	0.273	1961 (64.5)	2017 (66.4)	0.039
Hispanic or Latino	130 (4.3)	1933 (5.0)	0.034	130 (4.3)	117 (3.8)	0.022

ASD, absolute standardized difference.

Values are mean ± standard deviation or number (%).

Patient demographics for the ndCCB and control cohorts are presented in Table 2. Before PSM, the ndCCB cohort included 684 patients, with 38,663 patients in the control group. After matching, each cohort included 684 patients. The average age in the ndCCB and control groups was 73.1 ± 11.4 years and 73.4 ± 11.1 years, respectively. Most patients in both groups were female (57.2% and 55.3%, respectively) and White (both 69.5%). The rate of essential hypertension was 58% in both cohorts after PSM.

**Table 2.** Demographics of Patients With POAG With and Without ndCCB Use Before and After PSM

	POAG With ndCCB (*N* = 684)	POAG Without ndCCB (*N* = 38,863)		POAG With ndCCB (*N* = 684)	POAG Without ndCCB (*N* = 684)	
Characteristics	Value Before PSM	Value Before PSM	ASD	Value After PSM	Value After PSM	ASD
Age at index, years	73.1 ± 11.4	68.5 ± 13.2	0.375	73.1 ± 11.4	73.4 ± 11.1	0.029
Essential hypertension	396 (57.9)	10,603 (27.3)	0.339	396 (57.9)	400 (58.5)	<0.001
Sex						
Female	391 (57.2)	18,807 (48.4)	0.176	391 (57.2)	378 (55.3)	0.038
Male	247 (36.1)	13,885 (35.7)	0.008	247 (36.1)	260 (38.0)	0.039
Race						
Black or African American	123 (18.0)	6,522 (16.8)	0.032	123 (18.0)	123 (18.0)	<0.001
White	451 (65.9)	18,047 (46.4)	0.401	451 (65.9)	451 (65.9)	<0.001
American Indian or Alaska Native	10 (1.5)	415 (1.1)	0.035	10 (1.5)	10 (1.5)	<0.001
Unknown	81 (11.8)	7,318 (18.8)	0.195	81 (11.8)	77 (11.3)	0.018
Native Hawaiian or other Pacific Islander	10 (1.5)	3,680 (9.5)	0.358	10 (1.5)	10 (1.5)	<0.001
Asian	15 (2.2)	1,466 (3.8)	0.093	15 (2.2)	12 (1.8)	0.032
Other	12 (1.8)	1413 (3.6)	0.116	12 (1.8)	17 (2.5)	0.051
Ethnicity						
Unknown	196 (28.7)	17,013 (43.8)	0.319	196 (28.7)	199 (29.1)	0.010
Not Hispanic or Latino	463 (67.7)	19,915 (51.2)	0.340	463 (67.7)	460 (67.3)	0.009
Hispanic or Latino	25 (3.7)	1,933 (5.0)	0.065	25 (3.7)	25 (3.7)	<0.001

Values are mean ± standard deviation or number (%).

### Progression to Severe POAG

**Figure 1.**
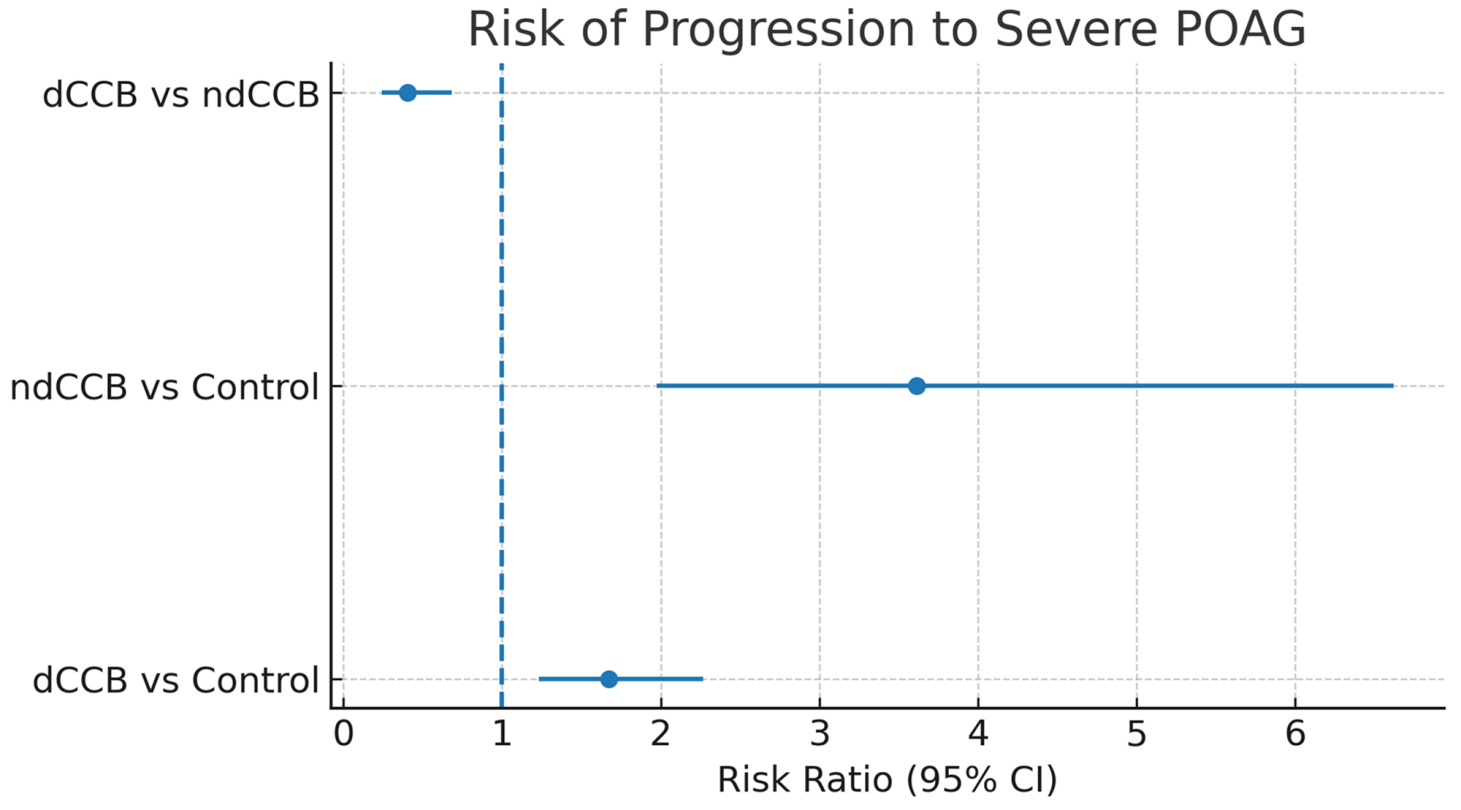
Forest plot of relative risk for progression from mild to moderate POAG to severe POAG after PSM. dCCB vs control: RR, 1.67 (95% CI, 1.23–2.27); ndCCB vs control: RR, 3.62 (95% CI, 1.97–6.62); dCCB vs ndCCB (680 vs 680): RR, 0.40 (95% CI, 0.24–0.68). Estimates derived from post-PSM counts shown in Table 3.

**Figure 2.**
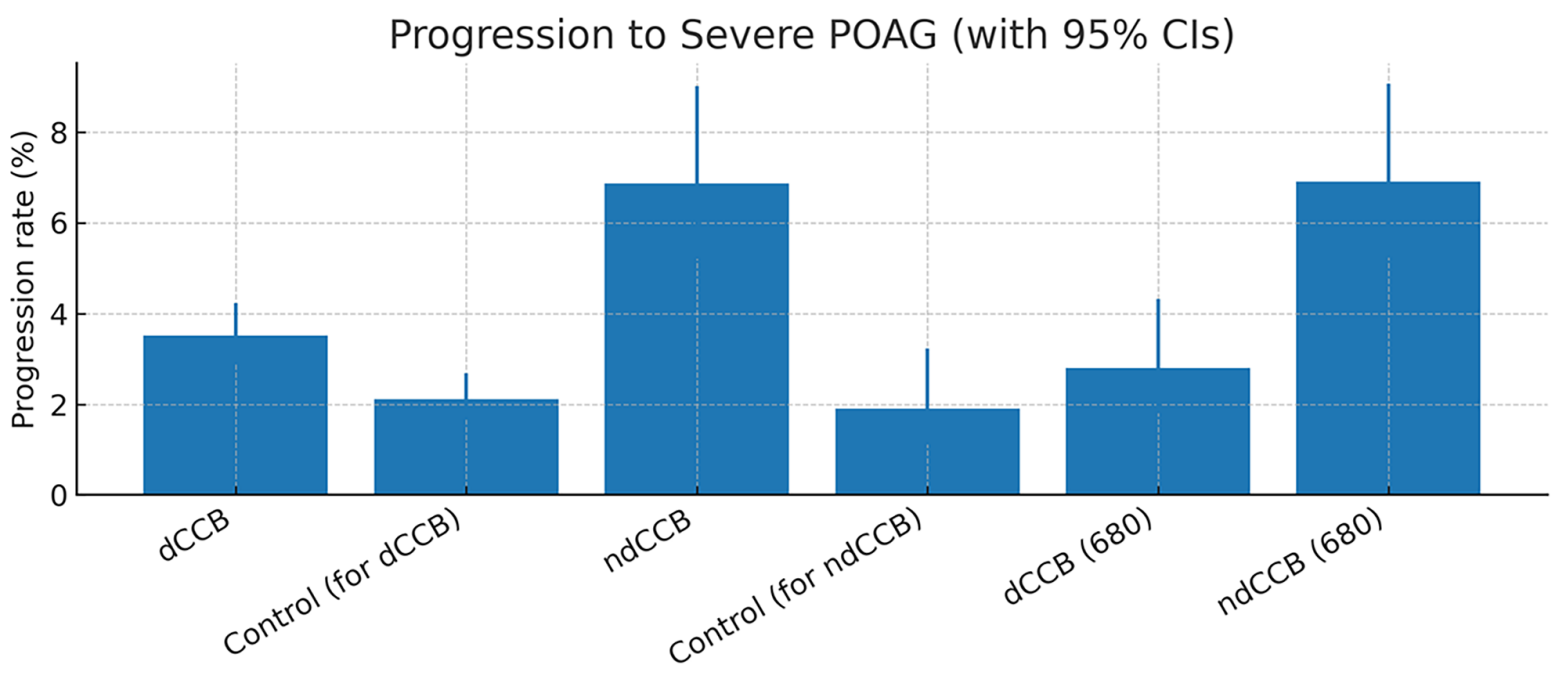
Progression rates (%) with 95% Wilson CIs for matched cohorts. dCCB vs its control: 3.5% vs 2.1%; ndCCB vs its control: 6.9% vs 1.9%; and dCCB vs ndCCB (680 vs 680): 2.8% vs 6.9%. Rates correspond with post-PSM counts in Table 3.

Results of intergroup comparisons are summarized in Table 3. Compared with patients with POAG not taking CCBs, patients in the dCCB cohort had a significantly increased risk of progression to severe POAG (RR, 1.67; 95% CI, 1.23–2.27) (Table 3; Fig. 1). Similarly, patients in the ndCCB cohort had a significantly increased risk of progression to severe POAG compared with patients with POAG not taking CCBs (RR, 3.62; 95% CI, 1.97–6.62) (Table 3; Fig. 1). When comparing the dCCB and ndCCB cohorts directly, patients in the dCCB group were associated with a significantly lower relative risk of glaucoma progression compared with patients using ndCCB (RR, 0.40; 95% CI, 0.24–0.68) (Table 3; Fig. 1). Progression proportions in the matched cohorts were 3.5% vs. 2.1% (dCCB vs. control), 6.9% vs. 1.9% (ndCCB vs. control), and 2.8% vs. 6.9% (dCCB vs. ndCCB) (Fig. 2).

**Table 3.** Incidence of Progression to Severe POAG in Patients Taking and Not Taking dCCBs

	No. of Patients	No. of Patients Progressing to Severe POAG	%
POAG with dCCB	3039	107	3.5
POAG without dCCB	3039	64	2.1
POAG with ndCCB	684	47	6.9
POAG without ndCCB	684	13	1.9
POAG with dCCB	680	19	2.8
POAG with ndCCB	680	47	6.9

## Discussion

In this study, patients receiving dCCB therapy demonstrated a significantly increased risk of progression to severe POAG (RR, 1.67; 95% CI, 1.23–2.27), whereas those treated with ndCCB exhibited an even greater risk (RR, 3.62; 95% CI, 1.97–6.62) compared with the non-CCB control cohort. Given that CCBs are widely prescribed for arterial hypertension, it is essential to consider the complex relationship between blood pressure regulation and glaucoma progression. The existing literature remains inconclusive, with some studies implicating elevated systolic blood pressure in glaucoma pathogenesis,^31^^,^^32^ whereas others indicate that reductions in diastolic blood pressure, which can result from antihypertensive treatment, may accelerate disease progression.^33^^,^^34^ Because CCBs exert their antihypertensive effects through reductions in vascular resistance and alterations in ocular blood flow, their potential contribution to the advancement of POAG warrants further investigation. These results underscore the need for a more comprehensive evaluation of antihypertensive pharmacotherapy in patients at risk for glaucomatous damage, particularly those with concurrent cardiovascular and ocular disease.

Previous studies have explored the association between CCB use and glaucoma development. Müskens et al.^4^ and Kastner et al.^13^ reported that patients using CCBs for hypertension management are more likely to develop glaucoma, aligning with our findings of increased POAG progression among CCB users. Similarly, Montesano et al.^35^ identified a link between CCB use and accelerated visual field deterioration in glaucoma patients. Tavakoli et al.^24^ explored the effects of various antihypertensive medications on glaucoma development and found that combining CCBs with nonselective beta-blockers was associated with POAG development, whereas ACE inhibitors, aldosterone antagonists, ARBs, and selective beta-blockers showed no such association. Conversely, Lee et al.^36^ reported that both CCBs and ARBs increased the risk of POAG, contradicting Iskedjian et al.,^37^ who suggested that antihypertensive medications, including CCBs and ACE inhibitors, may reduce the need for adjunctive glaucoma therapies. Gelmers et al.^38^ supported this protective hypothesis, proposing that CCBs may mitigate POAG progression through their anti-α1 adrenergic effects on ciliary arteries. In contrast, Vergroesen et al.^14^ found that ndCCBs increased glaucoma risk, opposing Tavakoli et al.’s findings,^24^ which implicated dCCBs instead. Our study reinforces the association between both dCCB and ndCCB use and an increased risk of POAG progression, highlighting the ongoing debate regarding the role of antihypertensive therapy in glaucoma pathophysiology.

Despite multiple studies linking CCB use with glaucoma progression, the underlying mechanisms remain unclear. Several theories have been proposed to explain this association. In vitro studies suggest that CCBs may decrease the response of extracellular matrix genes in lamina cribrosa cells under mechanical strain.^39^ Other research indicates that CCB-induced vasodilation may divert blood flow, particularly in ischemic tissues, potentially exacerbating glaucomatous damage.^38^ Additionally, IOP-independent mechanisms of glaucomatous neurodegeneration have been implicated. Kastner et al.^13^ reported that CCB use was associated with thinning of the macular ganglion cell–inner plexiform layer and macular retinal nerve fiber layer. Similar controversies exist regarding the impact of antihypertensive medications on low-tension glaucoma. Funk et al.^6^ found that CCBs accelerate disease progression in low-tension glaucoma, whereas Netland et al.^26^ reported a neuroprotective effect of CCBs in low-tension glaucoma patients with no evidence of progression in those patients compared with POAG patients. With POAG patients, they found no significant differences in the rate of progression, whether they were taking CCBs or not. This result might be explained by the neuroprotective effect of CCBs through the increase of blood supply related to blocking of T-type channels, which are more abundant in neuronal tissue. Whereas beta-blockers have been reported to offer some protection in POAG,^14^^,^^15^ other studies have linked them to low-tension glaucoma progression.^40^^,^^41^

Despite the strengths of this study, including a large and diverse patient population derived from a real-world dataset, several limitations should be acknowledged. The analysis relied on the accuracy of ICD-10 coding, which may be subject to misclassification, particularly regarding the severity of glaucoma, the documentation of CCB use, and the timeline for progression to severe glaucoma. Adherence to medication, control of arterial hypertension, and the follow-up duration, dosage, and duration of use of CCB, which can affect glaucoma progression, are also limitations of this study that cannot be assessed through the database. In addition to these limitations, the database does not show the baseline IOP, axial length, and glaucoma procedures performed, which might affect the outcome and rate of progression of glaucoma. Other risk factors, such as diabetes mellitus and obesity, were not matched between cohorts, which might affect the progression and control of glaucoma, which is considered a limitation of our study.

Residual immortal-time bias may persist despite restricting outcomes to those occurring after the index date and excluding patients with severe POAG before exposure, because patient-level person-time was unavailable and a full new-user, active-comparator design could not be implemented.^29^^,^^30^ Accordingly, our estimates are based on proportions rather than incidence rates or hazards and should be interpreted cautiously. In addition, Kaplan-Meier methods were not used because event times were unavailable and competing risks could not be handled appropriately.^42^^,^^43^ Future work should emulate a target trial with patient-level time-to-event data to directly estimate hazards and cumulative incidence using appropriate competing-risk methods.^29^

## Conclusions

Although prior studies have presented inconsistent findings, our results demonstrate that both dCCB and ndCCB use are associated with approximately a two-fold increased risk of progression to severe glaucoma among patients with mild to moderate POAG. These findings warrant further investigation to elucidate the underlying mechanisms linking CCB exposure to glaucoma progression. In addition, CCBs should be recognized as a risk factor, and patients taking CCBs should be followed more frequently to identify early progression.
